# Differential lipidomics of HK-2 cells and exosomes under high glucose stimulation

**DOI:** 10.7150/ijms.67326

**Published:** 2022-01-24

**Authors:** Weidong Wang, Tingting Li, Zhijie Li, Hongmiao Wang, Xiaodan Liu

**Affiliations:** Department of Nephrology, The First Affiliated Hospital of China Medical University, 155 North Nanjing Street, Shenyang, Liaoning, P.R. China, 110001

**Keywords:** Targeted lipidomics, Renal tubules, Exosomes, Diabetic kidney disease

## Abstract

Abnormal cellular lipid metabolism has a very important role in the occurrence and progression of diabetic kidney disease (DKD). However, the lipid composition and differential expression by high glucose stimulation of renal tubular cells and their exosomes, which is a vital part of the development of DKD, are largely unknown. In this study, based on targeted lipid analysis by isotope labeling and tandem mass spectrometry, a total of 421 and 218 lipid species were quantified in HK-2 cells and exosomes, respectively. More importantly, results showed that GM3 d18:1/22:0, GM3 d18:1/16:0, GM3 d18:0/16:0, GM3 d18:1/22:1 were significantly increased, while LPE18:1, LPE, CL66:4 (16:1), BMP36:3, CL70:7 (16:1), CL74:8 (16:1) were significantly decreased in high glucose-stimulated HK-2 cells. Also, PG36:1, FFA22:5, PC38:3, SM d18:1/16:1, CE-16:1, CE-18:3, CE-20:5, and CE-22:6 were significantly increased, while GM3 d18:1/24:1, GM3 were significantly decreased in exosomes secreted by high glucose-stimulated HK-2 cells. Furthermore, TAG, PC, CL were decreased significantly in the exosomes comparing with the HK-2 cells, and LPA18:2, LPI22:5, PG32:2, FFA16:1, GM3 d18:1/18:1, GM3 d18:1/20:1, GM3 d18:0/20:0, PC40:6p, TAG52:1(18:1), TAG52:0(18:0), CE-20:5, CE-20:4, CE-22:6 were only found in exosomes. In addition, the expression of PI4P in HK-2 cells decreased under a high glucose state. These data may be useful to provide new targets for exploring the mechanisms of DKD.

## Introduction

Lipid metabolic disorders participate in the occurrence and development of diabetic kidney disease (DKD) 1, 2. Changes in cellular lipid composition in the kidney under a high glucose state can cause the increase of active oxygen production, mitochondrial dysfunction and even induce apoptosis 3, 4, 5, 6. Although numerous researchers have acknowledged the important role of renal tubules in the development and progression of DKD, few studies have addressed overall lipid expression in renal tubules or the changes under high glucose stimulation 7, 8. Therefore, it is of great importance to get comprehensive information on lipid in renal tubules, thus further elucidating the role of lipid in the pathogenesis of DKD.

Over recent years, the role of the exosomes in the development of DKD has attracted increasing attention. Exosomes that can cooperate with the autophagy-lysosome pathway to relieve intracellular stress have an important role in maintaining cellular homeostasis 9, 10. Meanwhile, they act as carriers for material transfer and information communication between cells 11. Recent studies have revealed that the crosstalk between the tubular cells and other renal cell types in the nephron might have an important role in the disease progression 12, 13. Although the lipid composition of exosomes can affect their properties, it has been studied to a lesser extent compared with the attention of protein and RNA 14. Since exosomes secreted by different cells vary in lipid types, contents, and functions, clarifying the lipid composition and differential expression of the exosomes secreted by renal tubules under a high glucose state would provide a theoretical basis to clarify the mechanism of exosomal communication concerning renal tubules.

Herein, we presented a differential lipidomic analysis of renal tubules and their exosomes under high glucose stimulation and detected differentially expressed lipid species of cardiolipin (CL), monosialodihexosyl ganglioside (GM3), and cholesteryl ester (CE). We also initially explored the role of phosphoinositides (PIPs) in exosomal production and observed the uptake of tubular exosomes by glomerular mesangial cells (GMCs).

## Methods

### Cell culture

The HK-2 human renal proximal tubular epithelial cell line was purchased from the Shanghai Institute for Biological Sciences Cell Resource Center. The HK-2 cells were cultured in normal glucose Dulbecco's Modified Eagle Medium/Nutrient Mixture F-12 (DMEM/F-12) that was supplemented with 10% fetal bovine serum (FBS; FSP500, ExCell). Normal glucose DMEM/F-12 was a 1:1 mixture of DMEM (11966025, Gibco) and Ham's F-12 (11765054, Gibco) that contained 5.56 mmol/L glucose. The cells were maintained at 37°C in a 5% CO_2_ incubator. Once 80% confluence was reached, the cells were harvested using the standard trypsin digestion procedure and passaged at a split ratio of 1:2. The passage cells were cultured with exosome-depleted FBS (CMS101.03, Cellmax) in 5.5 mmol·L^-1^ glucose as the blank control group (NG) and 30 mmol·L^-1^ glucose as high glucose group (HG) for 48 hours.

### Exosomes isolation

Differential ultracentrifugation was used to extract exosomes from the HK-2 cells culture supernatants. In brief, HK-2 cells culture supernatants were collected and sequentially centrifuged at 2000×g, 4℃ for 10 min, and then at 10,000×g, 4℃ for 30 min to remove cells, cellular debris, and large vesicles. Supernatant fractions were filtered using 0.22-μm pore size filters. Filtered samples were then subjected to ultracentrifugation at 110,000×g for 75 min, twice at 4℃ to pellet the exosomes. The resulting exosomes were re-suspended in a small amount of phosphate buffer solution (PBS) and stored at -80℃.

### Nanoparticle tracking analysis (NTA)

Exosomes were thawed in a 25℃ water bath and placed on ice, after which they were directly diluted with 1×PBS for detection with tunable resistive pulse sensing (TRPS). Analyses of size distribution and concentration of exosomes were performed using qNano Gold with Izon Control Suite 3.3.2 from Izon Science.

### Protein determination and western blotting

Approx. 100μl premixed cracking solution (RIPA cracking solution and 1% PMSF inhibitor) was added to the cell and exosome samples separately. After cracking for 30min, they were centrifuged at 12,000×g, 4℃ for 20min. The supernatant was taken, and the protein concentrations were determined by using bicinchoninic acid (BCA; Beyotime) protein assay kit according to the manufacturer's instructions. Bovine serum albumin was used as standard protein.

Protein samples were fractionated by sodium-dodecyl-sulfate polyacrylamide gel electrophoresis (SDS-PAGE). After being transferred onto a PVDF membrane (Millipore), they were incubated with various primary antibodies, anti-CD63 (EXOAB-CD63A-1, SBI; 1:1000), anti-ALIX (YT6283, Immunoway; 1:1000), anti-Calnexin (YT0613, Immunoway; 1:1000), anti-P62 (P0067, Sigma; 1:1000), and anti-LC3 (L7543, Sigma; 1:1000), followed by goat anti-rabbit or mice Ig secondary antibodies (180202-001, SBI; 1:5000). Specific bands were detected using enhanced chemiluminescent (ECL; Beyotime) substrate. β-actin (BM0627, Boster) was used as an internal control. Relative band intensity was measured by using ImageJ software.

### Transmission electron microscopy (TEM)

5μl sample was dropped to the copper net and incubated at room temperature for 5min. A drop of 2% uranium peroxide acetate was added to the copper net and incubated at room temperature for 1 min. It was then dried for about 20min at room temperature and observed under nano-transmission electron microscope (Tecnai G2 Spirit BioTwin, FEI).

### Targeted lipidomics

Lipids were extracted using a modified version of Bligh and Dyer's method 15. All lipidomic analyses were carried out on an Exion ultra-performance liquid chromatography (UPLC) coupled with a SCIEX QTRAP 6500 PLUS system. UPLC-MS/MS analyses were conducted in electrospray ionization (ESI) mode, with following conditions: curtain gas = 20, ion spray voltage = 5,500 V, temperature = 400℃, ion source gas 1 = 35, and ion source gas 2 = 35. Polar lipids were separated using a Phenomenex Luna 3 µm of silica column (inner diameter 150 × 2.0 mm) with two mobile phases: mobile phase A (chloroform: methanol: ammonium hydroxide, 89.5: 10: 0.5) and mobile phase B (chloroform: methanol: ammonium hydroxide: water, 55: 39: 0.5: 5.5). Gradient separation was conducted as follows: the gradient was maintained with 95% A for 5 min and then linearly reduced to 60% in 7 min and held for 4 min, after which it declined to 30% and was then held for 15 min. Finally, the original gradient was applied and maintained for 5 min. Mass spectrometric multi-reaction monitoring (MRM) was established for various lipid identification and quantitative analysis 16, 17. Individual polar lipid species were quantified by referencing to spiked internal standards of the same lipid class including PE-d31(16:0/18:1), DMPE; PG-d31(16:0/18:1), DMPG; PI-d31(16:0/18:1), di-C8-PI; PS-d31(16:0/18:1), DMPS; PA-d31(16:0/18:1), PA(17:0/17:0); BMP-(14:0/14:0); LPE-17:1; LPI-17:1; LPS-17:1; LPA-17:0; GM3 d18:1/18:0-d3; d31-16:0, d8-20:4; PC-d31(16:0/18:1), DMPC; SM d18:1/d31-16:0, SM d18:1/12:0; LPC-17:0; Cer d18:1-d7/15:0; Sph-d18:1/17:0; CL 22:1(3)-14:1.

Glycerol lipids, including diacylglycerols (DAGs) and triacylglycerols (TAGs) were quantified using a modified version of reversed-phase HPLC/MS/MS**.** Separation of neutral lipids was achieved on a Phenomenex Kinetex-C18 2.6 µm column (i.d. 4.6x100 mm) using a mobile phase containing chloroform: methanol: 0.1 M ammonium acetate (100:100:4), at a flow rate of 160 µl/min. d5-TAG (16:0)3; d5-TAG (14:0)3; d5-TAG (18:0)3 were used to individual spike TAG. d5-DAG (1,3-17:0); d5-DAG (1,3-18:1) were used to spike individual DAG based on Neutral miss MS/MS technology.

Free cholesterols, sterols and their esters were analyzed by HPLC-MS/MS, conducted in atmospheric pressure chemical ionization (APCI) mode, with Cholesteryl-2,2,3,4,4,6-d_6_ Octadeconate; Cholesterol-26,26,26,27,27,27-d6 as internal standards.

Chloroform: methanol: (1:1), 2.4 M hydrochloric acid and 0.25 M EDTA were added to the sample, followed by grinding at low temperature. The sample was centrifuged after incubation for 3 hours at 4°C, after which the lower organic phase was extracted. Chloroform was added for the second extraction, followed by washing organic phase using 1N hydrochloric acid: methanol (1:1). The organic phase was dried in a vacuum rotary concentrator. As previously reported, 40% methylamine: water: n-butanol: methanol (36:8:9:47) was used for deacylation and analyzed by Thermo ICS-5000 ion chromatography. PI4P, PI3P, PI4P, PI(3, 4)P2, PI(3, 5)P2, PI(4, 5)P2, and PI(3, 4,5) P3 were used to build an external calibration curve for quantitative analysis. The analysis was supported by Lipidall Technologies Company Limited.

### Fluorescence microscopy

To visualize the uptake of exosomes into GMCs (Sicencell), an equal quantity of exosomes secreted by HK-2 cells with or without high glucose stimulation were both labeled with PKH67 (mini67, Sigma) according to the manufacturer's instructions. Labeled exosomes were cultured with GMCs for 24h. Confluent GMCs were washed three times with PBS, after which the cells were fixed with 4% paraformaldehyde and kept out of light for 30min. Cells were washed three times with PBS and stained with DAPI (28718903, Solarbio) for 4-6min. After washing three times, confluent GMCs were photographed by using Nikon C2 confocal microscope.

### Statistical analysis

GraphPad 7.0 was applied for statistical analysis and mapping. Data are expressed as mean ± SEM. Student's t-test was used for comparison between two groups. A *P-*value < 0.05 was considered as statistical significance.

## Results

### HK-2 cells lipid composition and differential expression under high glucose stimulation

**Fig 1:**
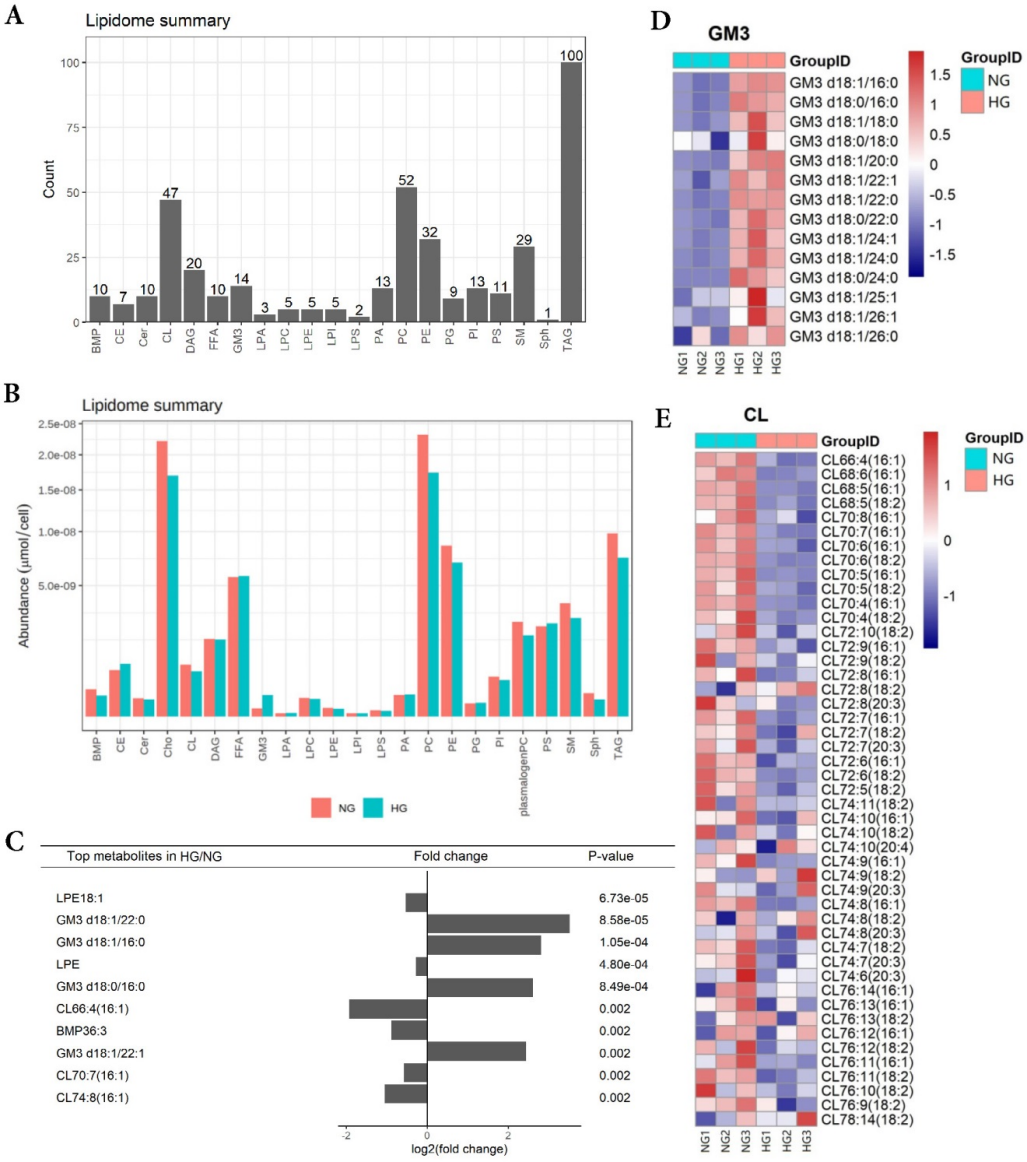
** HK-2 cells lipid composition and differential expression under high glucose stimulation**. **A** Species detected in each 21 major lipids of HK-2 cell. **B** Average lipid content of each 21 major lipids in HK-2 cells. **C** The expression of GM3 d18:1/22:0, GM3 d18:1/16:0, GM3 d18:0/16:0, GM3 d18:1/22:1 were significantly increased in HG, while the expression of LPE18:1, LPE, CL66:4(16:1), BMP36:3, CL70:7(16:1), CL74:8(16:1) were significantly decreased in HG. **D** The heatmap of GM3. The expression of GM3 d18:1/22:0, GM3 d18:1/16:0, GM3 d18:0/16:0, and GM3 d18:1/22:1 were all significantly increased in HG. **E** The heatmap of CL. The expression of CL66:4(16:1), CL70:7(16:1), and CL74:8(16:1) were all significantly decreased in HG. CL, cardiolipin; GM3, monosialodihexosyl ganglioside; NG, control group; HG, high glucose group.

In order to obtain lipid composition and differential expression profiles of HK-2 cells stimulated by high glucose, we performed a quantitative analysis of lipid expression of three parallel samples by isotope labeling and tandem mass spectrometry. First, we detected 21 major lipids in HK-2 cells, including TAG, PC, CL et al., in which TAG identified the most species (100) (Figure 1A). The average content of each lipid was detected, revealing the average PC content to be highest in both NG and HG groups (Figure 1B). The results showed that GM3 d18:1/22:0, GM3 d18:1/16:0, GM3 d18:0/16:0, GM3 d18:1/22:1 expressions were significantly elevated in high-glucose-treated HK-2 cells, while the LPE18:1, LPE, CL66:4(16:1), BMP36:3, CL70:7(16:1), and CL74:8(16:1) expressions were significantly lowered. Also, among these, LPE18:1, GM3 d18:1/22:0, GM3 d18:1/16:0, and LPE were the first four metabolites with the most significant differences between groups (Figure 1C and E).

### Exosomes lipid composition and differential expression under high glucose stimulation

**Fig 2:**
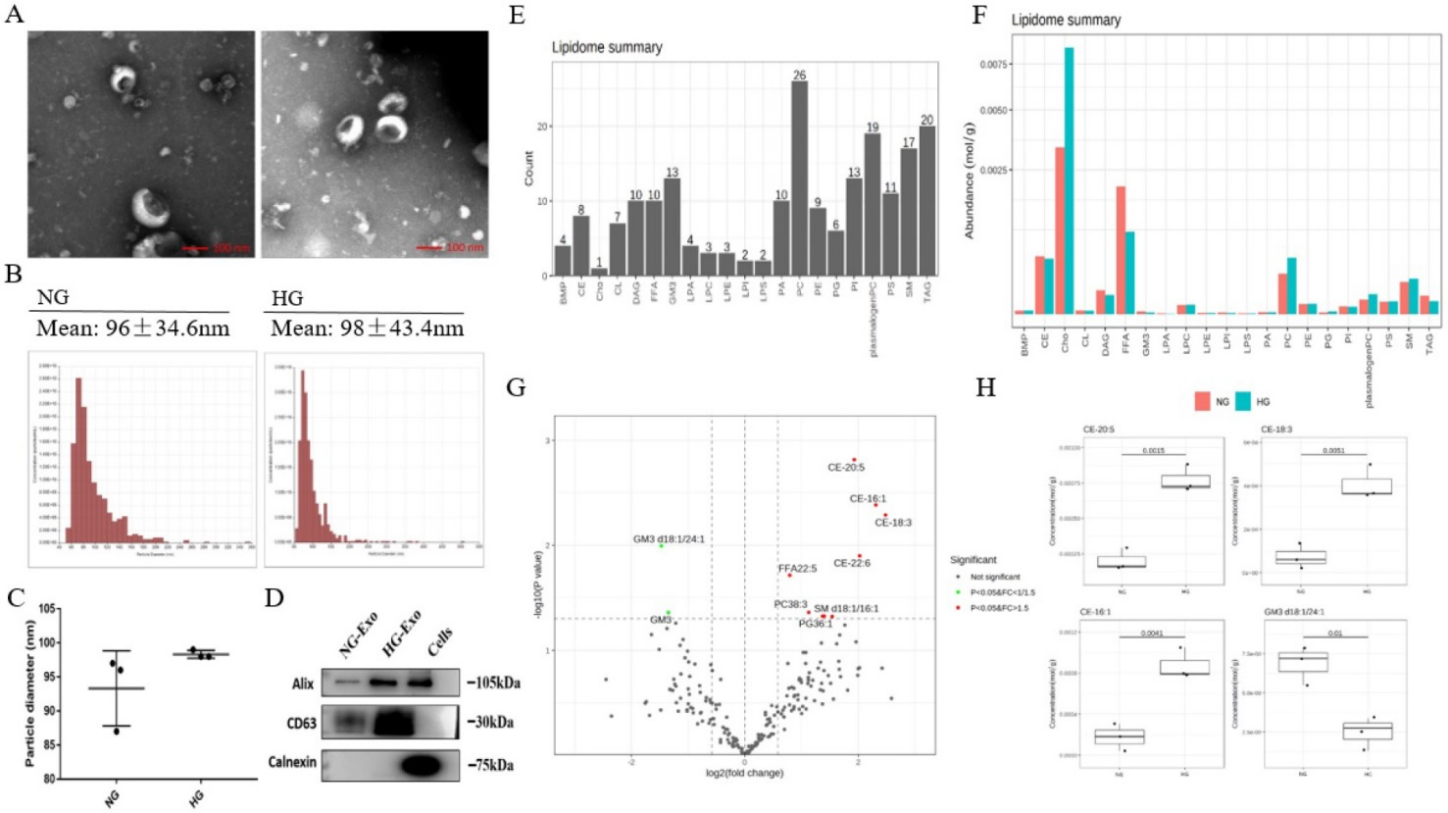
** Exosomes lipid composition and differential expression under high glucose stimulation**. **A** Representative electron micrograph images of exosomes secreted by HK-2 cells. Scale bar, 100nm. **B, C** Size distribution of exosomes secreted by HK-2 cells using NTA. **D** Western blotting of exosomal markers (including Alix and CD63) and endoplasmic reticulum molecular chaperon Calnexind in exosomes secreted by HK-2 cells, cells used as control. **E** Species detected in each 21 major lipids of exosomes secreted by HK-2 cells. **F** Average lipid content of each 21 major lipids in exosomes secreted by HK-2 cells. **G** In exosomes secreted by HK-2 cells, the expression of PG36:1, FFA22:5, PC38:3, SM d18:1/16:1, CE-16:1, CE-18:3, CE-20:5, CE-22:6 were significantly increased in HG, while the expression of GM3 d18:1/24:1, GM3 were significantly decreased in HG. **H** CE-20:5, CE-16:1, CE-18:3, GM3 d18:1/24:1 were the first four metabolites with the most significant differences between HG and NG groups. NG, control group; HG, high glucose group; NG-Exo, exosomes secreted by NG; HG-Exo, exosomes secreted by HG.

In order to analyze the lipid composition of exosomes secreted by HK-2 cells, exosomes were purified from the culture medium of HK-2 cells. TEM showed the typical structure and size of exosomes (Figure 2A). The size distribution of exosomes was detected by NTA. The average diameters were 93.3nm and 98.3nm in NG and HG, respectively, and there were no significant differences between the two groups (Figure 2B and C). Western blotting showed that the exosomes were positive for Alix and CD63 while negative for Calnexin (Figure 2D).

Next, we performed the quantitative detection of lipid expression of the exosomes. We detected 21 major lipids, including CE, Cho, PC et al., in which PC identified the most species (26) (Figure 2E). The average content of each lipid was detected, and the average Cho content was highest in both NG and HG groups (Figure 2F). We further compared the differential expression. Results showed that PG36:1, FFA22:5, PC38:3, SM d18:1/16:1, CE-16:1, CE-18:3, CE-20:5, CE-22:6 were significantly increased in HG, while GM3 d18:1/24:1, and GM3 were significantly decreased. Also, among these, CE-20:5, CE-16:1, CE-18:3, and GM3 d18:1/24:1 were the first four metabolites with the most significant differences between the two groups (Figure 2G and H).

In addition, the lipid composition of HK-2 cells and exosomes was different, with TAG, PC, and CL being significantly decreased in the latter, while there was no change in FFA, PI, PS, and other lipids varied less. A total of 421 and 218 lipid species were quantified in HK-2 cells and exosomes, respectively, revealing about 205 species (47.24%) common for the two samples. 13 (3%) lipid species were attained only in exosomes, including LPA18:2, LPI22:5, PG32:2, FFA16:1, GM3 d18:1/18:1, GM3 d18:1/20:1, GM3 d18:0/20:0, PC40:6p, TAG52:1(18:1), TAG52:0(18:0), CE-20:5, CE-20:4, CE-22:6.

### PIPs, exosomes and autophagy

**Fig 3:**
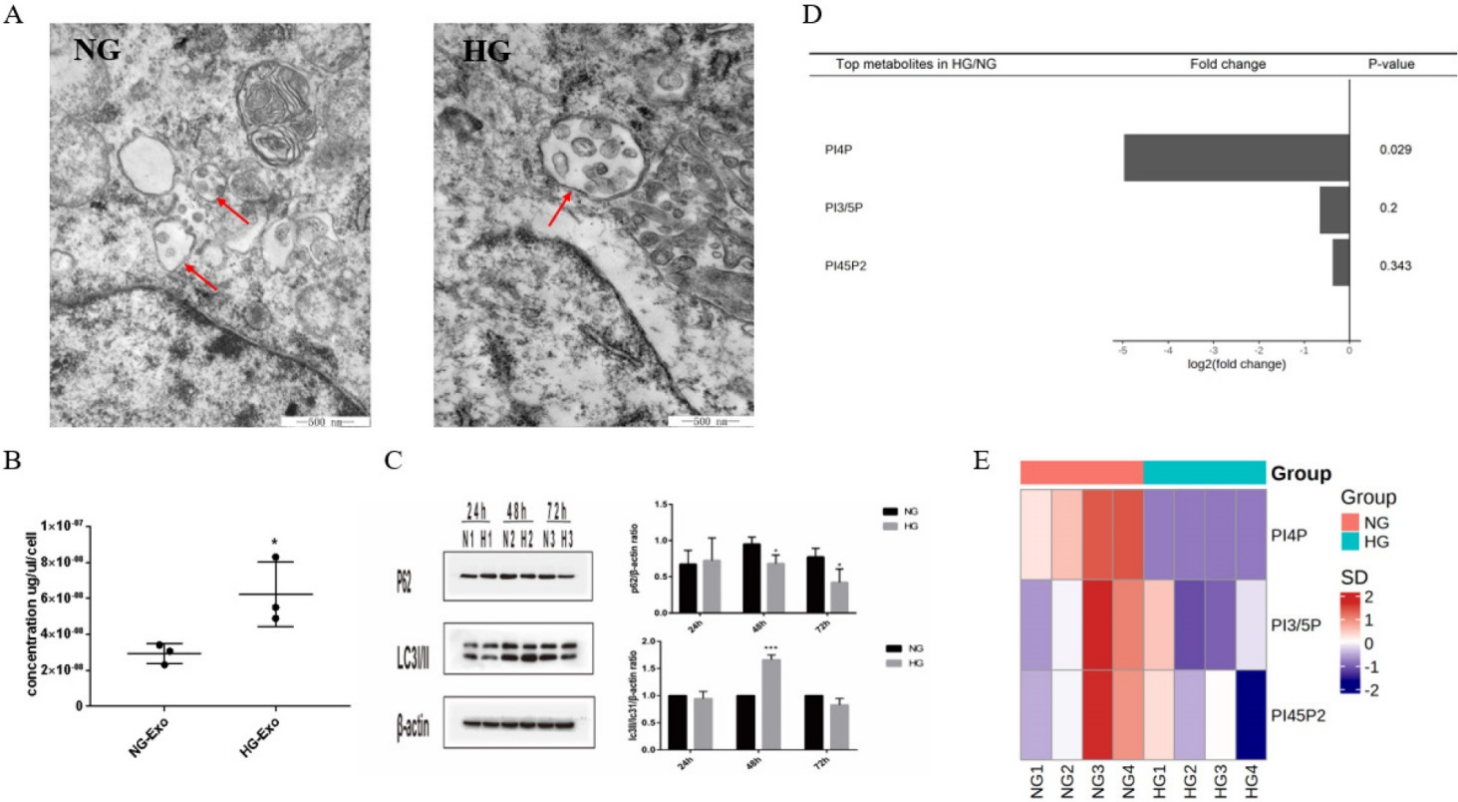
** Differential expression of exosomes, autophagy and PIPs in HK-2 cells under high glucose stimulation. A** Changes in volume of cellular MVBs in HK-2 cells after high glucose stimulation for 48h by transmission electron microscopy (the arrows show the MVBs). **B** Changes of the amount of exosomes protein secreted by HK-2 cells after high glucose stimulation for 48h by BCA analysis.** C** Changes of the expression of LC3 and P62 in HK-2 cells after high glucose stimulation by western blotting. **D** Differential expression of PIPs in HK-2 cells after high glucose stimulation for 48h. **E** The heatmap of PIPs. PIPs, Phosphoinositides; NG, control group; HG, high glucose group; NG-Exo, exosomes secreted by NG; HG-Exo, exosomes secreted by HG. ^*^P<0.05, ^***^P<0.001.

PIPs have been found to have an important role in maintaining intracellular homeostasis by regulating both the expressions of exosomes and autophagy through the transformation of multivesicular bodies (MVBs). To initially explore the possible correlation between exosome release and autophagy pathways regulated by PIPs in tubular cells, we detected the expression levels of exosomes, autophagy, and PIPs in HK-2 cells under high glucose stimulation. The TEM results showed an increased volume of cellular MVBs in the HG group, while the number of precursors of exosomes in MVBs also increased (Figure 3A). The protein concentration of exosomes determined by BCA was significantly higher in the HG group than in the normal control group, indicating that high glucose promotes the exosomes production of HK-2 cells (Figure 3B). The expression levels of autophagy-related proteins LC3 and P62 measured by western blotting showed autophagy activation of HK-2 cells under high glucose stimulation (Figure 3C). PIPs changes were observed by isotope labeling and tandem mass spectrometry, and a significant decrease in PI4P expression was found after high glucose treatment in HK-2 cells (Figure 3D and E).

### Uptake of HK-2 cells-derived exosomes by GMCs

**Fig 4:**
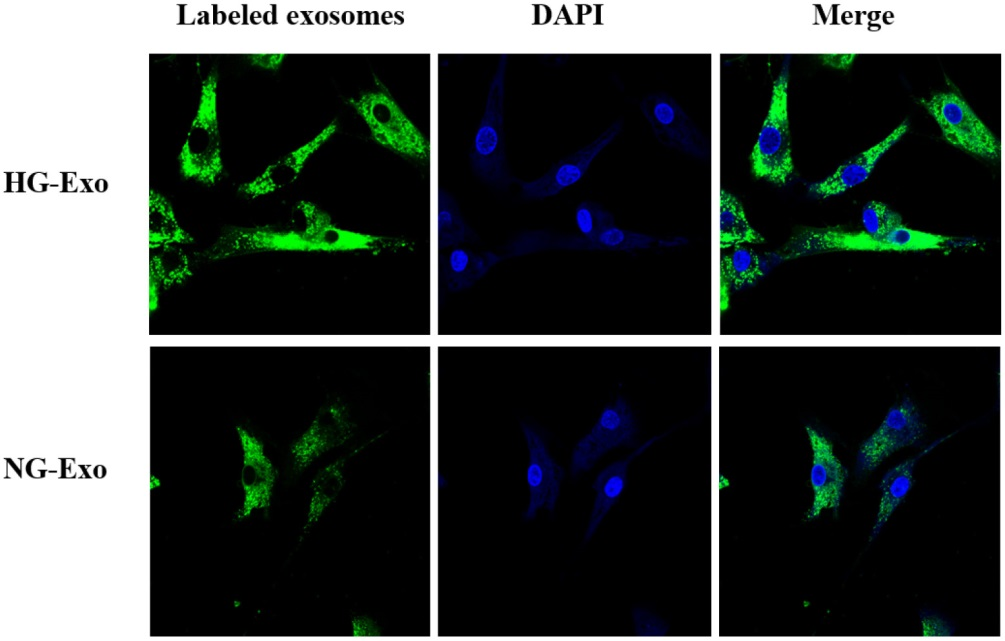
** Uptake of HK-2 cells-derived exosomes by GMCs.** HK-2 cells-derived exosomes were fluorescently labeled with PKH67 (green), then incubated with GMCs. Cell nuclei were stained with DAPI (blue). A confocal microscope was used to visualize the uptake of labeled exosomes by GMCs. GMCs, glomerular mesangial cells; NG, control group; HG, high glucose group; NG-Exo, exosomes secreted by NG; HG-Exo, exosomes secreted by HG.

Figure 4 shows the uptake of HK-2 cells-derived exosomes with or without high glucose stimulation by GMCs, respectively. Isolated exosomes were fluorescently labeled and co-incubated with GMCs. Confocal microscopy showed that exosomes were distributed around the nucleus and in the cytoplasm of GMCs, which confirmed the uptake of HK-2 cells-derived exosomes by GMCs.

## Discussion

Our study provides the differentially expressed lipid species of HK-2 cells and exosomes under high glucose stimulation, which were not previously reported. Interestingly, species of GM3, such as GM3 d18:1/22:0, GM3 d18:1/16:0, GM3 d18:0/16:0, GM3 d18:1/22:1 were significantly increased in high glucose-stimulated HK-2 cells, and GM3 d18:1/24:1, GM3 were significantly decreased in their exosomes. In addition, GM3 d18:1/18:1, GM3 d18:1/20:1, GM3 d18:0/20:0 were only found in exosomes. GM3 is the most widely distributed ganglioside in the body, consisting of glucose, galactose, and sialic acid characterized by sialic groups associated with the ceramide skeleton structure 18. It is involved in the regulation of a variety of cell functions, including signaling transduction, proliferation, differentiation, and apoptosis. According to recent studies, the serum concentration of GM3 is higher in patients with type 2 diabetes, hyperlipidemia, and obese patients, while GM3 can promote the removal of insulin receptors and reduce insulin signaling 19. Our results indicated elevated levels of all differential expressed GM3 species in HK-2 cells under high glucose stimulation, especially the increase of GM3 with lateral chain (22: 0) that was the most obvious. Zhang *et al.* 20 also observed elevated GM3 d18:1/22:0 expression in the kidney cortex of diabetic nephropathy mice, which was consistent with our results. The study of streptozotocin-induced type 2 diabetes rats conducted by Anela *et al.* 21 found unchanged GM3 in the glomeruli but significantly increased GM3 expression in renal tubules, which is in line with our finding of GM3 changes in renal tubules. Kwak *et al.* 22 found a GM3 decrease in the glomerular content of streptozotocin induced diabetic rats, followed by a loss of charge selective filtration barrier in the glomeruli. It is important to note that GM3's role in the glomeruli and renal tubules may vary. GM3 is also an important part of lipid raft 23. Changes in GM3 expression levels in lipid raft alter the activities of Na^+^-glucose cotransporter type 2 (SGLT2) and renal Na^+^/K^+^/Cl^-^ cotransporter, which are both lipid raft-dependent 24. Some researchers presumed that a higher concentration of GM3 increases SGLT2 and Na^+^/K^+^/Cl^-^ cotransporter activities, leading to increased tubular reabsorption and efferent arteriole hydrostatic pressure and then causing renal tubular cell injury by damaging the tubuloglomerular balance. Kumari* et al.* 25 found that GM3 in urinary exosomes of patients showed significant difference between the DKD and diabetes mellitus. Taken together, the role of GM3's elevated expression under high glucose in renal tubules should be addressed by future research.

Importantly, our results also showed that some species of CL, including CL66:4(16:1), CL70:7(16:1), and CL74:8(16:1) were significantly decreased in high glucose-stimulated HK-2 cells. Compared with HK-2 cells, CL was significantly decreased in the exosomes. CL is located almost entirely in the mitochondrial inner membrane and is an important part of maintaining the mitochondrial structure. It has an important role in electron conduction, adenosine triphosphate production, energy metabolism, and apoptosis 26, 27. In diabetes, mitochondrial morphological changes and dysfunction are often accompanied by pathological CL change 28,29. The proximal renal tubular epithelial cells require enough ATP to maintain its active transport and reabsorption of various substances. As high energy-consuming cells, proximal renal tubular epithelial cells are rich in mitochondria 30. Mitochondrial fragmentation has been observed in proximal tubular epithelial cells in early diabetes. Zhang *et al.* 31 found that CL was essential for maintaining tubular mitochondrial function in mice with type 1 diabetes. Our results may provide a new theoretical basis for studying the mitochondrial injury mechanism of DKD.

Exosomes have an important role in maintaining cellular homeostasis by cooperating with the autophagy-lysosome pathway. Previous studies have found that changes in PIPs expression levels could affect both exosome secretion and cellular autophagy by regulating the transformation of MVBs 32, 33, 34. Nina *et al.* 35 found that in PC-3 cells, inhibition of PIKfyve, which substrate is PI3P, increased secretion of exosomes and induced secretory autophagy, thus showing that these pathways were closely linked by PIPs. Based on the above-mentioned, we observed the changes of PIPs, exosomal production, and autophagy in high glucose-stimulated HK-2 cells. Our results showed that high glucose stimulated both the secretion of exosomes and the activation of autophagy in HK-2 cells, while a significant decrease was found in PI4P expression. Further studies are necessary to investigate the exosome-autophagy regulatory mechanism of PIPs involved in high glucose stimulation.

As the messenger, exosomes have an important role in intracellular signaling and functional changes in the development of DKD 36, 37, 38. We fluorescently labeled HK-2 cells-released exosomes and demonstrated their paracrine uptake by GMCs. Compared with their mother cells, exosomes have a special composition, which is making them to be more stable and functional due to the rich cholesterol, sphingolipids, saturated phospholipids, and lipid raft domains 39. Even small changes in lipid composition can affect the properties of the membrane, thus having a great effect on their function. In the present study, we found that TAG, PC, CL was significantly decreased in exosomes compared with HK-2 mother cells, while 13 species of lipids including LPA18:2, LPI22:5, PG32:2, FFA16:1, GM3 d18:1/18:1, GM3 d18:1/20:1, GM3 d18:0/20:0, PC40:6p, TAG52:1(18:1), TAG52:0(18:0), CE-20:5, CE-20:4, CE-22:6 were detected only in exosomes. The work presented here provides a basis for further analysis of the paracrine roles of exosomes released by tubular cells.

## Conclusions

In conclusion, our study demonstrated the importance of lipidomics analysis in providing new targets to explore the pathogenesis of DKD. Further studies on the function of the differential expressed lipids as well as the comprehensive lipid studies on other cells in the nephron and their exosomes may provide a new perspective that would further elucidate the mechanism of DKD.

## Abbreviations

DAG: Diacylglycerol
TAG: Triacylglycerol
PC: Phosphatidylcholine
CL: Cardiolipin
GM3: Monosialodihexosyl ganglioside
PE: Phosphatidylethanolamine
BMP: Bis (monoacylglycerol) phosphate
CE: Cholesteryl ester
Cer: Ceramide
FFA: Free fatty acid
PA: Phosphatidic acid
PG: Phosphatidylglycerol
PI: Phosphatidylinositol
PS: Phosphatidylserine
LPA: Lysophosphatidic acid
LPC: Lysophosphatidylcholine
LPE: Lysophosphatidylethanolamine
LPI: Lysophosphatidylinositol
LPS: Lysophosphatidylserine
SM: Sphingomyelin
Sph: Sphingosine
Cho: Cholesterol
PIPs: Phosphoinositides
